# The effect of venovenous extra-corporeal membrane oxygenation (ECMO) therapy on immune inflammatory response of cerebral tissues in porcine model

**DOI:** 10.1186/1749-8090-8-186

**Published:** 2013-08-29

**Authors:** Qiyi Chen, Wenkui Yu, Jiangliang Shi, Juanhong Shen, Yimin Hu, Tao Gao, Juanjuan Zhang, Fengchan Xi, Jianfeng Gong, Jieshou Li, Ning Li

**Affiliations:** 1 Department of General Surgery, Jinling Hospital, Medical School of Nanjing University, Nanjing 210002, Jiangsu, China

**Keywords:** Extra-corporeal membrane oxygenation, Immune inflammatory response, Neurological impairment, Adult respiratory distress syndrome

## Abstract

**Background:**

Extra-Corporeal Membrane Oxygenation (ECMO) therapy is associated with high risk of neurologic injury. But the mechanism of neurologic injury during and/or after ECMO therapy is still unclear. Recent animal experiments confirmed that ECMO treatment increases the immune inflammatory response. The aim of this study is to investigate the effect of VV- ECMO on immune inflammatory response of cerebral tissues and neurological impairment.

**Methods:**

18 porcine were randomly divided into control, sham and ECMO group (n = 6/group). ECMO was run 24 h in the ECMO group, and serum collected at 0, 2, 6, 12 and 24 h during ECMO treatment for the analysis of cytokine (IL-1β, IL-6, IL-10, TNF-a) and cerebral injury specific biomarker S100B and NSE. After 24 h ECMO treatment, all animals were euthanized and cerebral tissues (hypothalamus, hippocampus and cortex) were collected for measure of mRNA and protein levels of cytokine (IL-1β, IL-6, IL-10, TNF-a).

**Results:**

The results during ECMO treatment showed that all the pro-inflammation cytokines were increased significantly after 2 h, and anti-inflammation IL-10 showed transient hoist in the first 2 h in serum. After 24 h ECMO therapy, the mRNA levels of pro-inflammation cytokines and anti-inflammation IL-10 were simultaneously up-regulated in cerebral tissues (hypothalamus, hippocampus and cortex). And protein concentrations also showed different increasing levels in cerebral tissues. However, during the ECMO treatment, S100B and NSE protein in serum did not change significantly.

**Conclusion:**

These findings suggest VV-ECMO treatment can not only lead to immune inflammatory response in blood, but can also produce immune and inflammatory response in cerebral tissues. However the extent of immune inflammation was not sufficient to cause significant neurological impairment in this study. But the correlation between cerebral inflammatory response and cerebral impairment need to further explore.

## Background

Since 1972, when Extra-Corporeal Membrane Oxygenation (ECMO) was, for the first time, reported as therapy for adult respiratory distress syndrome (ARDS) successfully [1], ECMO has been considered as an effective means of treatment for the severe ARDS patients, and the success rate is between 53 to 76% [2-5]. In ARDS patients, 70-100% of survivors exhibit a distinctive neurological impairment [6,7]. Hypoxia and immune inflammatory response is a key factor for ARDS patients’ neurological impairment [6,7]. Therefore, in theory, ECMO possibly improves cerebral dysfunction in ARDS patients by increasing oxygen supply to cerebral tissues. The recent ECMO therapy’s successful application to cardiopulmonary and brain resuscitation procedures proved that it may have a protective effect on brain function [8].

However, ECMO therapy is associated with high risk of neurologic injury. Poor neurologic outcomes have been reported in as many as 10- 60% of survivors [9-11]. But the mechanism of neurologic injury during and/or after ECMO therapy is still unclear. More recent animal experiments confirmed that after two hours of ECMO treatment, blood and tissues (liver, lung, intestinal and renal) inflammatory cytokines’ (IL-1β, IL-6, IL-8, TNF-a) expression was significantly increased [12]. Another experimental study showed that ECMO treatment can lead to damage of the intestinal mucosal barrier, bacterial translocation, and increased systemic immune inflammatory response [13]. However, it is uncertain whether ECMO treatment can lead to immune inflammatory response as peripheral in cerebral tissues.

Once these inflammatory cytokines are produced in the brain, or if there is a high expression of these inflammatory cytokines in the brain itself, they will damage the cerebral tissues. For example, this can lead to nerve cell apoptosis and necrosis [14,15], glial cells, neurons, axonal injury, and brain tissue edema [16,17], neurotransmitter transporter obstacles [18], the destruction of the blood–brain barrier [15]and increased oxidative stress injury of the brain tissue [19]and so on. This may be disastrous for ARDS patients. Therefore, in this study, the effect of the venovenous (V-V) ECMO therapy on cerebral tissues’ immune inflammatory response will be explored and we will further investigate whether the immune inflammatory response can cause neurologic injury by normal porcine model.

## Methods

### Animal preparation

This study was approved by the Animal Care Committee of Jingling Hospital. Twelve piglets of either sex weighed (30.1 ± 2.8) kg. Before induction of anesthesia, the animals received ketamine (20 mg/kgIM), diazepam (8 mg/kgIM), and atropine (0.1 mg/kg IM). Later ketamine (10-20 mg/kg/hr IV) and diazepam (8 mg/kg/hr IV) were infused to maintain anesthesia.

### Mechanical ventilation strategy

After performing tracheotomy and placing an internal diameter 6.0-cm tracheal tube, mechanical ventilation was established using volume-controlled mode with an FIO2 of 0.21 and the positive end-expiratory pressure was set at 5 mmHg.Tidal volumes were adjusted to 5-8 mL/kg at 15 breath/min.

### Establishment of the catheter and grouping

A 16G venous catheter was placed into the left internal jugular vein to administer Ringer’s lactate at a rate of 3 ml/kg/hr initially. The rate was increased to maintain the mean arterial pressure above 60 mmHg. A 16G catheter was positioned into the right femoral artery to monitor blood pressure.

After baseline measurements, animals were randomly divided into three groups: Control group, Sham group (to verify whether the required ECMO operative procedure affected the results) and ECMO group (VV-ECMO, n = 6/group). After heparin (150 U/kg IV) was given as a bolus to the sham and ECMO groups, a 14F Biomedicus venous drainage cannula (MedtronicPerfusion Systems, Minneapolis, MN) was inserted into the right femoral vein. Another 14F Biomedicus arterial cannula (MedtronicPerfusion Systems, Minneapolis, MN) was used for venous infusion and inserted into the internal jugular vein. Heparin was infused to keep the whole blood activated clotting time (ACT) at 180-220 s. Placement of cannula was confirmed by ultrasonograph.

### Experimental protocol

In the sham group, the vascular venous cannula was occluded at 0 hour. The VV-ECMO was established at 0 hour. VV-ECMO procedure is as follows: The ECMO circuit (Quadrox PLS, Maquet, Germany) was primed with 500 ml Voluven and 200-300 ml Ringer’s lactate. The venovenous-ECMO system consisted of a centrifugal pump (Rota flow Console, Maquet, Germany), and a heat exchanger (Heater-Cooler Unit HCU 30, Maquet,Germany) maintaining temperature at 37°C. Sweep gas was 100% oxygen at a flow rate equal to the blood flow rate (1:1). Blood in the circuit was drained from the right femoral vein and infused into the right internal jugular vein at the rate of 50 ml/kg^-1^min^-1^.

### Sample collection

Blood samples (at 0, 2, 6, 12, 24 hours during ECMO running) were collected from the right femoral artery for measurement of TNF-a, IL-1beta, IL-6, IL-10, S100B and NSE. Blood serum was centrifuged at 2500 rpm for 15 min at 4°C, and the serum was stored at −70°C.

For cerebral tissues’ cytokine measurement, all animals were euthanized with a bolus injection of potassium chloride (40 ml, 0.1 g/ml) at 24 hours of ECMO treatment. Frontal cortex, hippocampus, and hypothalamus were dissected on ice. All brain regions were extracted in 1 mL extraction buffer/100 mg tissue. Cerebral tissue samples were homogenized in an ice-cold lysis buffer containing 137 mM NaCl, 20 mM Tris – HCl (pH 8.0), 1% NP40, 10% glycerol, 1 mM PMSF 10 μ g/mL aprotinin, 1 μ g/mL leupetin, and 0.5 mM sodium vanadate. The tissue homogenate solutions were centrifuged with 14000 × g for 25 min at 4°C. The supernatants were collected and stored at − 80°C until analysis.

### Measurement of protein levels of cytokines, S100B and NSE in serum and cerebral tissues

Quantification of serum and cerebral tissues TNF-a, IL-1beta, IL-6, IL-10, S100B and NSE protein levels were assessed by ELISA kit (R&D Systems, Munich, Germany), according to manufacturer’s instructions. Cytokine results, reported as pg/mL of serum or per gram of tissue (pg/g) were expressed as mean values ± SD. Where indicated, cytokine amounts were also normalized to protein content. In this case, the cytokine concentration of total protein in the brain extracts was measured by Bradford assay (BioRad Laboratories). S100B and NSE reported as μg/L were expressed as mean values ± SD.

### Real-time PCR RNA preparation and analysis of expression of cytokine genes in cerebral tissues

At 24 h ECMO treatment, frontal cortex, hippocampus, and hypothalamus were collected and stored at − 80°C until analysis. The total RNA from the sorted cerebral tissues was isolated using Trizol reagent. Once isolated, 5 μg of total RNA was reverse transcribed to yield cDNA. For each sample, 1 μl cDNA was added to a 50 μl PCR containing 0.5 μl primer, 8 μl SYBR green I premix and 11.3 μl ddH2O. The PCR temperature profile consisted of a single cycle at 95°C for 10 min; 40 cycles for 15 s at 95°C, for 15 s at 60°C, and for 20 s at 72°C (extension), and a final cycle at 72°C for 2 min. Real-time quantitative PCR was performed using a Rotor Gene 3000 system (Corbet, Australia). Gene expression was analyzed using the Rotor-Gene Real-Time Analysis Software 6.1. *GAPDH* was used as an internal control gene in order to normalize the PCRs for the amount of RNA added to the reverse transcription reactions. The primer sequences are shown in Table 1.

**Table 1:** **The primer sequences of the cytokine** (**TNF**-**a**, **IL**-**1β**, **IL**-**6 and IL**-**10**)

	**Forward**	**Reverse**
**TNF**-**a**	5’-TTGCCAGAGGGAGACCCCCG-3’	5’-CGGGCAGGTTGATCTCGGCA-3
**IL**-**1β**	5’- GGAAACTCCAAAGGCCGCCA-3’	5’- GCTTCGGGGTTCTTCAGCCCA-3’
**IL**-**6**	5’-ACAAATGCCGGCCTGCTGGA-3’	5’- ATGCCCGTGGACGGCATCAA-3’
**IL**-**10**	5’- TGCCCCACATGCTCCGGGAA-3’	5’-CCGGTCAGCAACAAGTCGCCC-3’

## Results

### TNF-a, IL-1β, IL-6 and IL-10 proteins concentrations in serum (Figure 1)

**Figure 1:**
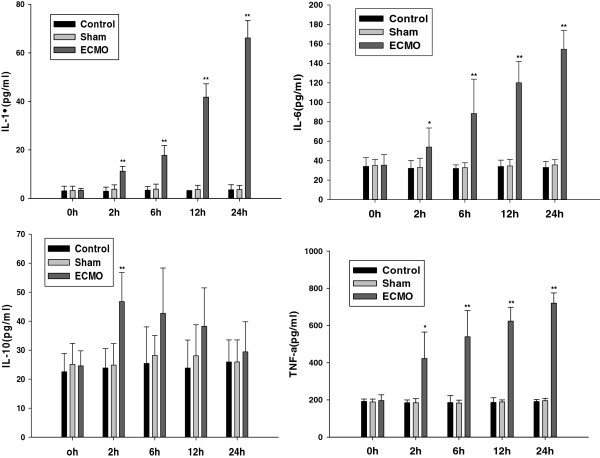
**Effects of ECMO therapy on cytokine (IL-1, IL-6, IL-10 and TNF-a) in serum.** The levels of cytokine (IL-1, IL-6, IL-10 and TNF-a) were assessed 0, 2 6, 12 and 24 hours post-ECMO treatment by using specific ELISA. Results are reported as histograms representing the cytokine mean concentrations with SD. Asterisks inside the graphs indicate the significance of comparison with control group: *P < 0.05, **P < 0.01.

Compared with the control group, the inflammatory cytokines (TNF-a, IL-1β, IL-6 and IL-10) did not show significant change in the sham operation group (at each time point, P > 0.05). After 2 h ECMO therapy, the number of pro-inflammatory cytokines TNF-a, IL-1β and IL-6 in the ECMO group was significantly higher than that of control group. Anti-inflammatory cytokine IL-10 showed a transient raise in the first two hours after ECMO treatment, and then decreased to normal levels gradually.

### TNF-a, IL-1β, IL-6 and IL-10 proteins concentrations in cerebral tissues (Figure 2)

**Figure 2:**
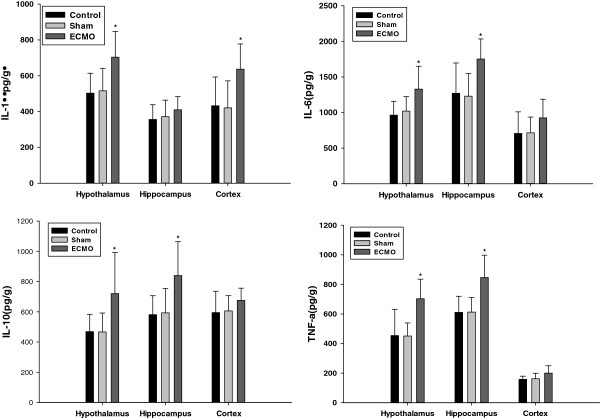
**Effects of ECMO therapy on cerebral tissues expression of cytokines at 24 hour’s post-ECMO treatment.** The levels of cytokine (IL-1, IL-6, IL-10 and TNF-a) were assessed 24 hours post-ECMO treatment by using specific ELISA, as described in the Methods section. Results are reported as histograms representing the cytokine mean concentrations with SD. Asterisks inside the graphs indicate the significance of comparison with control group: *P < 0.05.

There was no statistical difference between the control and sham groups (p > 0.05). After 24 h ECMO treatment, pro-inflammation cytokine IL-1β, IL-6, TNF-a, and anti-inflammation cytokine IL-10 protein concentrations in the hypothalamus were notably higher than those of the control group (p < 0.05). Although there was no change in the hippocampus IL-1βprotein concentrations in the control and ECMO groups (p > 0.05), the IL-6, IL-10 and TNF-a showed a substantial increase (p < 0.05). In the cortex region, only IL-1βprotein concentrations were higher than that of the control group (p < 0.05).

### TNF-a, IL-1β, IL-6 and IL-10 mRNA expression in cerebral tissues (Figure 3)

**Figure 3:**
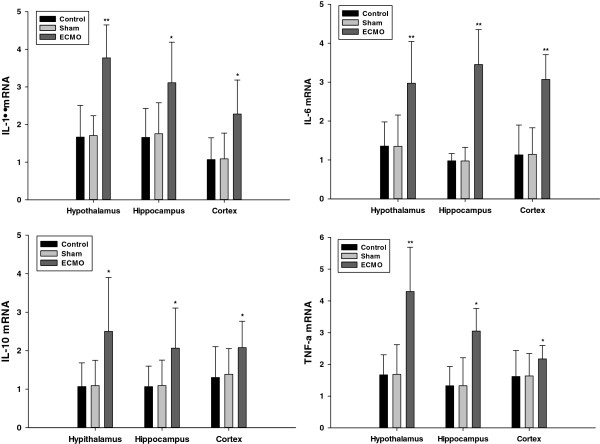
**Effects of ECMO therapy on cerebral tissues mRNA expression of cytokines after 24 hour’s ECMO treatment.** The mRNA levels of cytokine (IL-1, IL-6, IL-10 and TNF-a) were assessed 24 hours post-ECMO treatment by Real-time PCR, as described in the Methods section. Results are reported as histograms representing the cytokine mRNA mean levels with SD. Asterisks inside the graphs indicate the significance of comparison with control group: *P < 0.05, **P < 0.01.

There was no statistical difference between the control and sham groups (p > 0.05). After ECMO treatment, all the cytokine expression of mRNA showed a significant up-regulation compared with the control group in the three brain regions.

### S100B and NSE concentrations proteins in serum (Figure 4)

**Figure 4:**
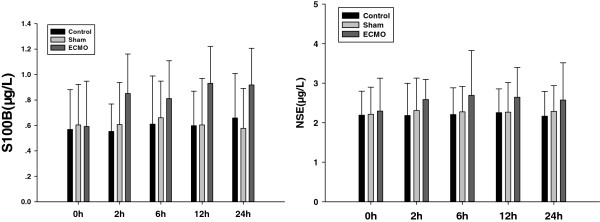
**Effects of ECMO therapy on S100B and NSE in serum.** The levels of S100B and NSE were assessed 0, 2 6, 12 and 24 hour’s ECMO treatment by using specific ELISA. Results are reported as histograms representing the S100B and NSE mean concentrations with SD.

There was no statistical difference between the control and sham groups for S100B and NSE (p > 0.05). During ECMO treatment, S100B and NSE protein increased slightly, but there was no statistical difference between the control and ECMO groups either (p > 0.05).

## Discussion

ECMO has been considered an effective means of therapy for the severe ARDS patients, and the efficiency rate has been between 53 and 76% [2-5]. Hypoxia is the main pathophysiological and physiological characteristic of ARDS that causes systemic organ injury. So ECMO therapy possibly improves high incidence of cerebral dysfunction in ARDS patients (70-100%) by increasing the oxygen supply to the brain tissue, which is most sensitive to hypoxia, theoretically. The recent successful application of ECMO therapy to cardiopulmonary and brain resuscitation procedures have proved that it may have a protective effect on brain function.

However, ECMO is associated with high risk of neurologic injury. Poor neurologic outcomes have been reported in as many as 10-60% of survivors [9]. The study reported that 58% patients developed clinical neurological syndrome including epilepsy, fatigue, pathological pupil and postpone sober [20], neuropsychological disorders and movement disorders [20], cerebral edema [20] and brain atrophy [10], 52% showed symptoms of neuroimaging abnormalities, and 41% of EEG abnormalities [11]. The mechanism of neurologic injury during and/or after ECMO therapy is still unclear.

Recent research reported that imbalance of immune inflammatory response is one of the focus issues of ECMO therapy. Due to various reasons like circulating blood exposure to foreign surface of ECMO circuit, blood flow shear stress, expression of cytokine and activation of complement system, abnormality of coagulation and fibrinolysis system etc., neutrophils are activated, ultimately resulting in the abnormal activation of systemic immune response [12,21]. In this experiment, we also observed that the peripheral serum pro-inflammatory cytokine TNF-α, IL-6 and IL-1β concentrations were raised rapidly after two hours during the ECMO treatment. And anti-inflammatory IL-10 presented a short term increase during the first two hours of ECMO. However, it is uncertain whether ECMO treatment can also lead to a peripheral immune inflammatory response in cerebral tissues.

Lots of investigations confirmed that peripheral circulating inflammatory cytokines such as TNF-a, IL-1β and IL-6 can invade into the brain through perventricular organ or transport mechanism of vascular endothelial cells [22-24]. These cytokines can also recruit other inflammatory mediators into brain tissue [25]. They can also injure the blood–brain barrier (BBB) and increase the permeability of BBB [24,26,27]. In our study, we found that after 24 h ECMO treatment, all of the pro-inflammation and anti-inflammation cytokine mRNA expressed were significantly up-regulated in the hypothalamus, the hippocampus and the cortex. The gene post-transcriptional levels of pro-inflammation cytokine IL-1β, IL-6, TNF-a and anti-inflammation cytokine IL-10 increased notably to different levels in the hypothalamus, or/and the hippocampus and the cortex. The results indicate that ECMO therapy can lead to immune inflammatory response of brain tissue.

Mountains of studies show that cytokine manifest toxicity on cerebral tissues. Brain ventricles or parenchyma injection of IL-1β may aggravate ischemic brain injury [28]. IL-1β and TNF-a, which inject into the brain, can lead to abnormal animal behavior [29]. Given IL-1β receptor antagonist (IL-1RA) will reduce the brain injury and death of the cranial nerves [30]. TNF-a and IL-1β can activate the hypothalamic-pituitary-adrenal (HPA) axis, the autonomic nervous system and the nucleus of neurons and glia, alter the expression of neurotransmitters, affect the permeability of cerebral blood vessels, and result in brain edema, brain cells apoptosis and brain tissue remodeling [18,31-34].

To investigate the effect of immune inflammatory response of cerebral tissues after ECMO therapy on neurological impairment, we measured the cerebral injury specific biomarker S100B and NSE (Neuron -specific Enolase) in serum [35,36]. S100B is most abundant in the glial cells of the central nervous system (CNS), mainly in astrocytes. Neuron-specific Enolase are present almost exclusively in the cytoplasm of neurons (γ–γ isoenzyme) and neuroendocrine cells (α–γ isoenzyme). Our data showed that ECMO treatment did not increase the concentrations of S100 and NSE in serum. This indicates that although the cerebral tissue appeared to develop immune inflammatory response during the ECMO procedure, it did not result in notable neurological impairment.

Our experimental findings seem to be in contradiction with the current studies (no significant neurological impairment versus poor neurologic outcomes). The main reasons include: first, in the study, the VV-ECMO bypass method was used rather than the venoarterial (V-A) ECMO method. Current studies believe the injuries were observed mainly in patients treated with VA-ECMO [10,11,37]. This is because in the VA-ECMO model rather than in the VV-ECMO model [11,37], the carotid artery needs to be ligated and nonpulsatile flow generated in the brain, which results in loss of autoregulation, hypoxia and ischemia in the brain. In addition, the blood is returned directly into the artery in VA-ECMO. However, the lungs function as a filter in the VV-ECMO model [11]. Secondly, in order to deplete the effect of the disease itself on the experimental results, normal pig model was used as the experiment subject. In other words, the neurological impairment may be related to the disease itself rather than to ECMO therapy. Thirdly, the increasing extent of pro-inflammatory cytokines is not sufficient to cause brain injury. At the same time, the anti-inflammatory cytokine IL-10 also increased in cerebral tissues. A number of studies confirmed that the anti-inflammatory cytokine IL-10 has a significant protective effect on brain injury [38,39].

In this study, there are some limitations. First, to investigate the effect of ECMO technology on brain injury, we used a normal pig model as the experiment subject. Although it can rule out the effect of the disease itself on the experimental results, it does not reflect the relationship of pathophysiological changes between various diseases and ECMO treatment. Secondly, this study did not check morphology alteration to determine brain injury. Although S100B and NSE are specific biomarkers for neurological injury, they cannot offer response to the extent of cerebral tissue injury.

## Conclusion

In conclusion, the results of our study showed VV-ECMO treatment can not only lead to immune inflammatory response in blood, but can also produce immune and inflammatory response in cerebral tissues. However the extent of immune inflammation was not sufficient to cause significant neurological impairment in this study. But the correlation between cerebral inflammatory response and cerebral impairment need to further explore. The study results may provide a basis for the development of treatment strategies for neurologic injury during and/or ECMO therapy in clinical practice. And the results of this study suggest that clinical ECMO treatment may need to be used in conjunction with an anti-inflammation therapy.

## Abbreviations

ECMO: Extra-corporeal membrane oxygenation; V-A: Venoarterial; V-V: Venovenous; BBB: Blood–brain barrier.

## Competing interests

The authors declare that they have no competing interests. This research was supported by National Natural Science Foundation (81270884),

Jiangsu Province Special Program of Medical science (BL2012006),

Grant for 12th five-year plan major project (AWS11J03), Grant for 12th five-year plan major project (WS12J001), Jiangsu Province's Key Medical Talent Program (RC2011128).

## Authors' contributions

QC, J Shi and J Shen participated in the collection of data. NL and WY conceived and designed this study. TG, JZ and FX did the statistical analysis. QC, YH and JG wrote the first draft of the paper and JL commented on the draft. All other authors provided comments and approved the final manuscript.
